# Complete Genome Sequence of *Methanolinea tarda* NOBI-1^T^, a Hydrogenotrophic Methanogen Isolated from Methanogenic Digester Sludge

**DOI:** 10.1128/genomeA.00876-14

**Published:** 2014-09-04

**Authors:** Kyosuke Yamamoto, Hideyuki Tamaki, Hinsby Cadillo-Quiroz, Hiroyuki Imachi, Nikos Kyrpides, Tanja Woyke, Lynne Goodwin, Stephen H. Zinder, Yoichi Kamagata, Wen-Tso Liu

**Affiliations:** a Bioproduction Research Institute, National Institute of Advanced Industrial Science and Technology (AIST), Higashi, Tsukuba, Ibaraki, Japan; b Department of Civil and Environmental Engineering, University of Illinois at Urbana-Champaign, Urbana, Illinois, USA; c School of life Sciences, Arizona State University, Tempe, Arizona, USA; d Swette Center for Environmental Biotechnology at the Biodesign Institute, Arizona State University, Tempe, Arizona, USA; e Department of Subsurface Geobiology Analysis and Research (D-SUGAR), Japan Agency for Marine-Earth Science & Technology (JAMSTEC), Yokosuka, Kanagawa, Japan; f DOE Joint Genome Institute, Walnut Creek, California, USA; g Department of Biological Sciences, Faculty of Science, King Abdulaziz University, Jeddah, Saudi Arabia; h Department of Microbiology, Cornell University, Ithaca, New York, USA

## Abstract

Here, we report a 2.0-Mb complete genome sequence of *Methanolinea tarda* NOBI-1^T^, a methanogenic archaeon isolated from an anaerobic digested sludge. This is the first genome report of the genus *Methanolinea* isolate belonging to the family *Methanoregulaceae*, a recently proposed novel family within the order *Methanomicrobiales*.

## GENOME ANNOUNCEMENT

*Methanolinea tarda* NOBI-1^T^, an H_2_/CO_2_-using methanogen, was isolated from mesophilic methanogenic sludge digesting municipal sewage in Nagaoka, Niigata Prefecture, Japan and described as a novel species of a novel genus within the order *Methanomicrobiales* (1). Based on 16S rRNA gene-based phylogeny, *M. tarda* NOBI-1^T^ belongs to the family level clade E1/E2, which is the outer group of the previously known three families. Recently, several strains belonging to the E1/E2 clade have been isolated and characterized, and a novel family *Methanoregulaceae* was proposed for the E1/E2 group as the fourth family within the order *Methanomicrobiales* (2). The family *Methanoregulaceae* is comprised of five isolated strains: *M. tarda* NOBI-1^T^, *Methanolinea mesophila* TNR^T^ (2), *Methanoregula boonei* 6A8^T^ (3), *Methanoregula formicica* SMSP^T^ (4), and *Methanosphaerula palustris* E1-9c^T^ (5). Since the natural habitats and physiological features of these *Methanoregulaceae* members vary by species, the taxonomic identification of the species relies largely on molecular phylogeny, and the characteristic genetic and physiological properties of this group distinguishable from other families remain largely unclear. The whole-genome sequence of *M. tarda* NOBI-1^T^ provides the first genomic information of the species belonging to the genus *Methanolinea* and will contribute to an improved understanding of the unique features of the family *Methanoregulaceae*.

The whole-genome shotgun sequencing was performed using a combined Illumina and Roche GS-FLX Titanium approach. Sequence assembly was carried out using the GS De Novo assembler Newbler (version 2.3). Manual finishing efforts raised the quality of the assembly to that of a finished genome. Genes were identified using Prodigal (6) as part of the JGI genome annotation pipeline (7), followed by a round of manual curation using the JGI GenePRIMP pipeline (8). Additional gene functional annotation and comparative analysis were performed within the Integrated Microbial Genomes (IMG-ER) platform (9).

The complete genome sequence length was 2,052,856 bp with a G+C content of 56.5%. The genome contains 2,057 protein-coding sequences, 55 pseudo genes, 46 tRNA genes, and an rRNA operon including one 23S large-subunit gene and one 16S small-subunit gene. A total of 77.5% of open reading frames (1,634) are protein-coding genes with function prediction.

Gene classification by the NCBI Clusters of Orthologous Groups (COG) categories (10) reveals that the genome harbors all of the genes involved in hydrogenotrophic methanogenesis pathway and genes for formate dehydrogenase, which are essential to utilize H_2_/CO_2_ and formate for growth and methane production. The genome contains one clustered regularly interspaced short palindromic repeat (CRISPR) locus and 9 CRISPR-associated genes, and 16 transposases and inactivated derivatives, suggesting the contribution of viral predation and gene transfer events by mobile genetic elements to the evolution of *M. tarda* NOBI-1^T^. Further comparative analyses with the genomes of other species belonging to the *Methanoregulaceae* and/or species within other taxa will provide insights into the unique genetic and physiological characteristics of the species within the *Methanoregulaceae* lineage.

### Nucleotide sequence accession numbers.

This whole-genome shotgun project has been deposited in DDBJ/EMBL/GenBank under the accession no. AGIY00000000. The version described in this paper is AGIY00000000.2.
